# Repurposing the drug, ivermectin, in COVID-19: toxicological points of view

**DOI:** 10.1186/s40001-022-00645-8

**Published:** 2022-02-05

**Authors:** Farshad M. Shirazi, Roya Mirzaei, Samaneh Nakhaee, Amir Nejatian, Shokouh Ghafari, Omid Mehrpour

**Affiliations:** 1 Arizona Poison & Drug Information Center, College of Pharmacy and University of Arizona College of Medicine, University of Arizona, Tucson, AZ USA; 2 Faculty of Pharmacy and Pharmaceutical Sciences, Islamic Azad University, Tehran Medical Sciences University (IAUTMU), Tehran, Iran; 3 Venom and Biotherapeutic Molecules Lab., Medical Biotechnology Department, Biotechnology Research Center, Pasteur Institute of Iran, 13169-43551 Tehran, Iran; 4 Medical Toxicology and Drug Abuse Research Center (MTDRC), Birjand University of Medical Sciences, Birjand, Iran; 5 Department of Civil Eng., Sharif University of Technology, Tehran, Iran; 6 Infectious Diseases Research Center, Birjand University of Medical Sciences, Birjand, Iran; 7 Data Science Institute, Southern Methodist University, Dallas, TX USA

**Keywords:** Ivermectin, COVID-19, SARS-CoV-2, Coronavirus disease 2019

## Abstract

The global COVID-19 pandemic has affected the world’s population by causing changes in behavior, such as social distancing, masking, restricting people’s movement, and evaluating existing medication as potential therapies. Many pre-existing medications such as tocilizumab, ivermectin, colchicine, interferon, and steroids have been evaluated for being repurposed to use for the treatment of COVID-19. None of these agents have been effective except for steroids and, to a lesser degree, tocilizumab. Ivermectin has been one of the suggested repurposed medications which exhibit an in vitro inhibitory activity on SARS-CoV-2 replication. The most recommended dose of ivermectin for the treatment of COVID-19 is 150–200 µg/kg twice daily. As ivermectin adoption for COVID-19 increased, the Food and Drug Administration (FDA) issued a warning on its use during the pandemic. However, the drug remains of interest to clinicians and has shown some promise in observational studies. This narrative reviews the toxicological profile and some potential therapeutic effects of ivermectin. Based on the current dose recommendation, ivermectin appears to be safe with minimum side effects. However, serious questions remain about the effectiveness of this drug in the treatment of patients with COVID-19.

## Introduction

In late 2019, the world faced a new life-threatening disease, the coronavirus disease 2019 (COVID-19), caused by the severe acute respiratory syndrome coronavirus 2 (SARS-CoV-2) [1]. The causative agent was first reported in Wuhan City, the capital of Hubei province in China. On 30 January, 2020, the World Health Organization (WHO) revealed COVID-19 as a public health emergency and an international issue [2, 3]. Surprisingly, a high number of new cases were detected worldwide in the first week of March, and COVID-19 established as a pandemic. As of 12 March, 2020, more than 125,000 confirmed cases and more than 4600 deaths were reported in 118 countries [2, 4]. The most common symptoms of this disease include cough, fever, fatigue, shortness of breath, pneumonia, and the common cold [5]. Also, some less frequent symptoms of SARS-CoV-2 infection are anosmia, dysgeusia, skin lesion, gastrointestinal symptoms, and headache [6–9].

Throughout the COVID-19 pandemic, many therapeutic agents have been repurposed and applied empirically and within clinical trials. Prophylactic medications for COVID-19 could have benefits. Furthermore, the efficacy of remdesivir, hydroxychloroquine, lopinavir/ritonavir, convalescent plasma, and monoclonal antibody therapy in decreasing mortality, when administered late in the course of the disease, is debated [10, 11]. Ivermectin is an exciting anti-parasitic medication that has received much attention recently [12].

In 2015, Japanese scientist, Satoshi Omura, was awarded the Nobel Prize in Physiology and Medicine for the discovery of ivermectin in 1974–1975 [13]. Ivermectin was first marketed as an anti-parasite drug by Merck Sharp and Dohme in 1981. It remains the world's leading anti-parasitic agent for livestock [14]. Some studies examined ivermectin's utility in COVID-19 patients. It has been reported that its administration reduced hospital stay and mortality of COVID-19 patients [15–17]. There is a need for compilation of the latest information on this drug from different corners of the world. Such data are likely to provide a better picture of the scope and limitations of ivermectin use in COVID-19. In this perspective review, ivermectin’s efficacy, and possible toxicity in the treatment of COVID-19 disease as a means to explore opportunities for expanded use of this drug are discussed.

### Pharmacodynamics and pharmacokinetic of ivermectin

Ivermectin inhibits the transmission of chemicals at nerve synapses using glutamate-containing anion channels or γ-aminobutyric acid-containing chloride channels. It stimulates γ-aminobutyric acid (GABA) secretion from the presynaptic nerve end and increases binding to the postsynaptic receptors [18]. Also, ivermectin is believed to exhibit an effect that involves inhibition of viral protein translocation in a complex with importin (IMPα/β1) into the nucleus. The infected cells release interferon (IFN) that binds to the IFN receptors in neighboring cells, alerting them to a viral attack. The IFN-I and IFN-III receptors then further activate members of the JAK-STAT family. After entering the host cell, the COVID-19 virus acts in a way so as to interfere with the host cell’s natural anti-viral response through the effect of interferon. The proteins of SARSCoV-2 such as ORF3a, ORF6, and NSP1 suppress IFN-I signaling [19, 20]. As a result, cells around the virus-infected cells “fail” to receive “protective IFN signals”, allowing the SARS-CoV-2 virus to multiply and spread without hindrance [21]. It has been observed that ivermectin can enhance the expression of some IFN-related genes, such as IFIT1, IFIT2, IF144, IRF9, ISG20, and OASL [22]. The SARS-CoV-2 pathogenicity is possibly involved in inhibition of type I interferon (IFN-I) immune signaling, resulting in reduced Janus Kinase (JAK)-Signal Transducer and Activator of Transcription 1 (STAT1) signaling and anti-viral response. In the absence of a virus, phosphorylation activates STAT1 and can interact with IFN regulatory factor 9, which produces the IFN-stimulated gene factor 3 transcription complexes. This complex gets into the nucleus and binds to the IFN-stimulated response element, promoting host’s anti-viral response [23–27]. Ivermectin has the potential to target and inhibit SARS-CoV-2 replication by blocking viral entry into the nucleus. Hence, the Signal Transducer and Activator of Transcription 1 (STAT1) signaling is unaltered by the antagonistic activity of SARS-CoV-2, and the anti-viral response takes place. In vitro studies have demonstrated that the enzyme, CYP3A4, primarily metabolizes ivermectin. Depending on the in vitro method used enzymes, CYP2E1 and CYP2D6 were also involved in ivermectin’s metabolism, albeit to a much lesser extent than CYP3A4. The results of in vitro studies with human liver microsomes indicate that clinically relevant concentrations of ivermectin do not significantly inhibit the metabolic activities of CYP3A4, CYP2C9, CYP2E1, and CYP2E1 [28, 29].

Liver metabolisms convert ivermectin to at least ten metabolites, most of them being hydroxylated and demethylated derivatives of the drug. The drug is mainly eliminated in the feces, and fecal excretion accounts for 90% of the dose administered, with less than 2% of the dose excreted in the urine. Bile is the main route of excretion, and its elimination half-life is around a day [30].

### Therapeutic use and dosage of ivermectin

Ivermectin is indicated for treating strongyloidiasis of the intestinal tract and onchocerciasis [31].

Onchocerciasis patients are generally treated as an annual oral dose (e.g., 150 or 200 µg/kg). Lymphatic filariasis is treated in endemic areas once a year (300–400 µg kg) or sometimes, twice a year (150–200 μg/kg) [31, 32]. Maximum doses of 2000 μg/kg are well tolerated in patients with parasitic infections [32, 33].

Analysis of data on severe adverse events (SAE) over 13 years in Africa revealed a cumulative incidence of 1 reported SAE per 800,000 reported treatments with ivermectin [34, 35]. No significant parasitic resistance to this drug has been observed in humans. Evidence suggests that ivermectin is a safe and effective anti-parasitic and anti-inflammatory agent that will continue to be used a main therapeutic course for parasitic infections for years to come [36]. It is believed that ivermectin has many cellular targets, and it has some anti-bacterial and anti-cancer activity [37]. It has also been observed through both in vitro and in vivo studies that ivermectin has some effects on several viruses. Several studies have shown that ivermectin might be helpful in treating COVID-19 patients at both mild–moderate and severe phases of the disease, and also as a potential prophylaxis [38–40].

### The possibility of effectiveness against SARS-COV-2

#### Biological plausibility

**Fig. 1:**
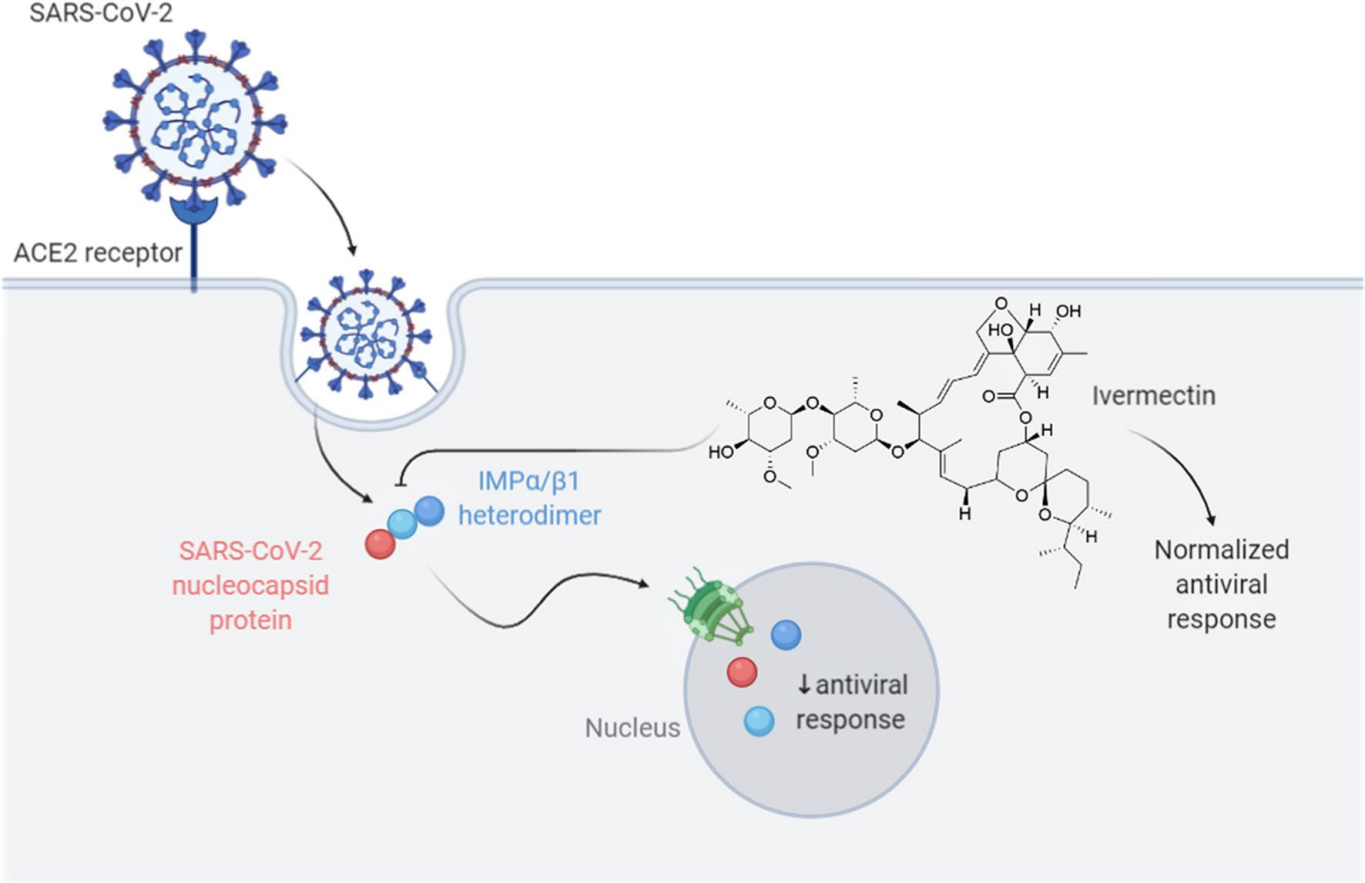
Proposed ivermectin mechanism of action on SARS-CoV-2. S protein on the viral envelope binds to the ACE-2 receptor and enters the cell through endocytosis, where IMPα/β1 binds to the viral nucleocapsid protein. The complex enters the nucleus through the nuclear pore complex (NPC) and separates, reducing the anti-viral reaction. In the presence of ivermectin, SARS-CoV-2 proteins are unable to bind to IMPα/ß1 heterodimers, as ivermectin destabilizes them

It is proposed that ivermectin might have anti-viral and immunomodulatory properties [16, 39, 40]. There are several biologically plausible reasons for the activity of ivermectin against SARS-cov2 in the treatment of COVID-19:

1. Ivermectin acts as a specific inhibitor of α/β-heterodimer in the nucleus of the cell, inhibiting replication of several RNA viruses [41–43]. It is presumed that ivermectin might inhibit SARS-CoV-2 using the same mechanism [44]. Additionally, Caly et al. have shown that the drug prevents SARS-CoV-2 replication and reproduction [44]. In their research, ivermectin was added to cells infected with SARS-CoV-2 RNA and they were analyzed by RT-PCR for observing the replication of the SARS-CoV-2 RNA at days 0–3. These cells were compared with the ones in the control group which did not receive ivermectin treatment. It was found that after 48 h, there was a ≈5000-fold reduction in viral RNA in the ivermectin treated cells compared to the control samples, and no toxicity was reported [44]. Spike protein on the viral envelope binds to the ACE-2 receptor and enters the cell through endocytosis, where importin (IMP) α/β1(IMPα/β1) binds to the viral nucleocapsid protein. The complex enters the nucleus through the nuclear pore complex (NPC) and separates, reducing the anti-viral reaction. In the presence of ivermectin, SARS-CoV-2 proteins are unable to bind to IMPα/ß1 heterodimers, as ivermectin destabilizes them (Fig. 1).
2. Ivermectin acts as a possible ionophore in infected cells disrupting biologic membrane in these cells [37].
3. Ivermectin has been reported to curb over-reacting innate and cellular immune responses during the inflammatory stage of COVID-19 [41, 45]. It has shown to have powerful anti-inflammatory properties through the inhibition of both production of cytokine and transcription of the most potent mediator of inflammation, the nuclear factor-κB (NF-κB) [46]. Therefore, theoretically, ivermectin might alleviate the symptoms of COVID-19 patients in the viral replication phase (in the first 7–10 days of infection) and later, in the hyper-inflammatory stage [41–44].
4. Interaction with ACE protein: Abdel-Mottaleb et al. demonstrated that ivermectin, hydroxychloroquine, and favipiravir are the most effective drugs that bind to ACE-2 and S protein in the human body [47]. A molecular modeling study by Dayer et al. also showed that ivermectin is one of the most effective agents that shields the host cells’ receptors from the SARS-CoV-2 spike protein [48]. Another study by Lehrer et al. showed that ivermectin docked leucine and histidine to the ACE-2 receptors [49]. In addition, Janabi et al., in computer-assisted molecular modeling, investigated the drug’s affinity for the active site of RNA-dependent RNA polymerase (RdRp). Five molecules, including ivermectin, were docked to the protein binding site using PyMol software, and the study reported a high binding affinity of ivermectin to RdRp [50].

### Clinical studies

In a multi-center case–control study of 280 hospitalized patients, ivermectin was administered as a single dose of 150 µg/kg to patients with COVID-19. It significantly reduced in-hospital mortality (ivermectin: 1.4% vs. non-ivermectin: 8.5%; HR: 0.2, CI 95%: 0.11–0.37, *p* < 0.0001) [51]. The findings of a double-blind randomized clinical trial (RCT) in Colombia do not support the ivermectin use in the treatment of mild COVID-19. In this study, patients with mild SARS-CoV-2 infection received ivermectin (300 μg/kg/day for 5 days, *n* = 200) or placebo (*n* = 200). They found that a 5-day course of ivermectin did not significantly improve the time taken for symptom resolution compared with the placebo [52]. In another retrospective study by Camprubí et al., 13 patients admitted with severe COVID-19 received a standard dose of ivermectin (200 μg/kg). No differences were reported in the mobility, microbiological or clinical outcomes in this group of patients compared to a similar group of patients who did not receive ivermectin [53]. In another randomized double-blind clinical trial, three groups of patients were administered ivermectin 24 mg, ivermectin 12 mg, and a placebo, respectively. The negative RT-PCR at day 5 failed to show statistical significances (24 mg ivermectin: 47.5%: 12 mg ivermectin, 35.0%; and placebo: 31.1%; *p*: 0.3). The decrease in viral load on day 5 was similar in the three groups [54]. Chaccour et al. in a small, double-blind RCT in Spain randomized 24 patients to ivermectin and placebo groups. They found no differences in positive RT-PCR on day 7, but they reported statistically significant reduction in the viral load [55]. WHO recently commissioned a meta-analysis to evaluate the clinical efficacy of ivermectin using the ACC Accelerator Program. The meta-analysis consisted of 18 RCTs evaluating doses up to 0.6 mg/kg of ivermectin on a total of 2282 RT-PCR positive patients with mild-severe COVID-19. Six trials evaluated the efficacy of ivermectin on survival in 1255 patients using doses between 200 and 400 µg/kg in a 1–5 day treatment and found a 75% reduction in mortality (Relative Risk: 0.25, CI 95% 0.12–0.52, *p* < 0.0002) with ivermectin treatment. In addition, a reduction in the hospital stay was reported. Regarding viral clearance, the effect of ivermectin was greater in clinical trials evaluating its effects for up to five days [56]. Mohan-Padhy et al. performed another meta-analysis on four studies with a total of 629 COVID-19 patients, to evaluate the therapeutic effects of ivermectin at a standard dose of 200 µg/kg as an adjuvant therapy to the standard care. An overall odds ratio of 0.53 (95% CI 0.29–0.96) was found for the all-cause mortality that was statistically significant (*p* = 0.04) [57]. In a pre-print paper, Maurya et al. argue that ivermectin in combination with doxycycline may enhance the inhibition of viral entry, therefore, decreasing viral load and replication [58]. A propensity-matched cohort study was conducted in Florida by Rajter et al. on 280 patients, 173 of whom were treated with ivermectin and 107 without it. The ivermectin group had a significantly lower mortality rate (13.3% vs. 24.5%, OR 0.47, CI 0.22–0.99, *p* < 0.05) [15]. Rahman et al. conducted a prospective study in Bangladesh, enrolling patients with mild-to-moderate COVID-19 disease and comparing the effect of ivermectin plus doxycycline to hydroxychloroquine plus azithromycin. Two hundred patients received ivermectin (18 mg on the first day) and doxycycline (100 mg twice daily for 5 days), while the other 200 patients received hydroxychloroquine (800 mg on the first day and 400 mg daily for the next 10 days) and azithromycin (500 mg on the first day and 250 mg daily for 4 days after that). From this study, ivermectin plus doxycycline together were found to be safe and more effective in early viral clearance in patients with mild-to-moderate disease compared to hydroxychloroquine and azithromycin combination [59]. Another clinical trial was conducted on in-patients with mild-to-moderate COVID-19 infections. Sixteen patients received a single dose of ivermectin of 200 µg/kg, hydroxychloroquine (HCQ), and azithromycin (AZT) on admission day. They were compared with 71 controls of matching age, gender, clinical features, and comorbidities who received only HCQ and AZT. [16 (100%) vs. 69 (97.2%)]. Two patients died in the controls. The average hospital stay was significantly lower in the ivermectin group than in the control group (7.62 ± 2.75 vs. 13.22 ± 5.90 days, *p* = 0.00005, size of effect = 0.82), and no toxicity or adverse events were noted [16]. In a cross-sectional study by Malik et al., most healthcare professionals were treated with either azithromycin or doxycycline, with favorable outcomes observed [60]. A further study by Alam et al. in Bangladesh reported that the combination of ivermectin and doxycycline is efficient in SARS-CoV-2 clearance in patients with mild-to-moderate disease. They enrolled 100 patients with mild and moderate COVID-19 in their study. They treated them with a combination of ivermectin (200 µg/kg single dose) and doxycycline (100 mg daily for 10 days) in addition to supportive treatment. All patients' symptoms improved within 72 h, no side effects were observed, no other treatment was required, and there was no death in this study [61].

Another clinical trial in Iraq was conducted with 70 COVID-19 patients (48 mild–moderate, 11 severe, and 11 critical patients) who were treated with 200 µg/kg of oral ivermectin per day for 2–3 days along with 100 mg PO doxycycline twice per day for 5–10 days plus standard therapy. The control group of COVID-19 patients (48 mild–moderate and 22 severe and zero critical patients) was given standard treatment [45]. The time to recovery was seen to have significantly reduced in the ivermectin–doxycycline group compared to the control group; the mean recovery time in the ivermectin–doxycycline group was 10.61 ± 5.3 days versus mean recovery time in a control group, 17.9 ± 6.8 days (*p* < 0.05). The mortality rate was 0/48 (0%), 0/11 (0%) and 2/11 (18.2%) in moderate, severe and critical COVID-19 patients in the ivermectin–doxycycline group, respectively, compared to 0/48 (0%) and 6/22 (27.27%) in moderate and severe COVID-19 patients in the standard therapy group, thus, showing no significant difference (*p* = 0.052). A preprint observational analysis in Argentina by Carvallo et al. has reported only one mortality rate in 167 COVID-19 patients (135 mild, 32 moderate to severe) who received a combination of dexamethasone, aspirin, enoxaparin, and ivermectin protocol [40]. Chowdhury et al. also compared the combination of ivermectin and doxycycline with hydroxychloroquine and azithromycin in people with mild-to-moderate COVID-19 infections. Patients were categorized into two groups. The first group (*n* = 60) received ivermectin (200 µg/kg one dose) and doxycycline (100 mg twice daily for 10 days), and the second group (*n* = 56) received hydroxychloroquine (400 mg on the first day and 200 mg twice daily for the next 9 days) and azithromycin (500 mg daily for 5 days). According to this study, ivermectin and doxycycline were found to be superior to hydroxychloroquine and azithromycin in treating mild-to-moderate COVID-19 patients, but the time required for the patients to be symptom-free and achieve a negative COVID test was not statistically significant [62]. Shouman conducted a randomized clinical trial in Egypt, including 340 (228 treated, 112 controlled) patients who received ivermectin twice on the day of a positive COVID-19 test and thereafter, once at day 3 based on their body weight (40–60 kg: 15 mg, 60–80 kg: 18 mg, > 80 kg: 24 mg). After a 2-week follow-up, the case group showed a significant reduction in COVID-19 symptoms compared to the control group (7.4% vs. 58.4%, *p* < 0.001) [63].

### The potential efficacy of ivermectin as prophylaxis for COVID-19

Héctor et al. conducted a prospective observational study in which they gave ivermectin and carrageenan daily to healthy volunteers for 28 days, comparing them to similarly healthy controls who did not take the drugs. Of the 229 study participants, 131 were treated with 0.2 mg ivermectin drops taken by mouth five times a day. After 28 days, none of the participants receiving ivermectin prophylaxis tested positive for SARS-COV-2, compared to 11.2% of the participants in the control group who tested positive [64]. In line with other prophylaxis reports, a recently published Preprint Matching Case–Control 374 study on medRxiv, which analyzed several drugs experimentally used as COVID-19 375 prophylaxis, showed a 73% reduction in COVID-19 infections in health care workers after two doses of ivermectin (OR 0.27; 95% CI 0.15–0.51) [65]. Remarkably, this study did not establish that a single dose of prophylaxis has a protective effect.

Probably the clearest evidence of the effectiveness of ivermectin as a prophylactic agent was recently published, comparing countries with presently active ivermectin mass drug delivery programs for the prevention of parasite infections. They found that COVID-19 case numbers were significantly lower in countries with such active programs [66]. What is evident is that both clinical and basic science studies are weak, and lack the preponderance of evidence in support of ivermectin for clinical use. However, ivermectin remains one of the drugs used for the treatment of COVID-19 and it has attracted our attention due to its safety profile.

### Safety, side effects, and tolerated doses of ivermectin in humans

In cases of accidental poisoning with veterinary ivermectin formulations in humans, symptoms like rash, edema, headaches, dizziness, nausea, vomiting, diarrhea, and asthenia were often reported. Other adverse effects include seizures, dyspnea, paresthesia, urticaria, abdominal pain, and contact dermatitis [67]. In humans, the most common adverse effects of ivermectin in oral doses are fatigue (13%) and headache (9%), dizziness and drowsiness (10%), and itching (12%) and lightheadedness (9%) [68]. Muñoz et al. assessed the adverse effects of ivermectin in 54 voluntary participants and reported that the most frequent side effects were headache (6.02%), dysmenorrhea (5.54%), throat pain (1.80%) and diarrhea (1.80%). Of the 33 reported adverse effects, 10 were classified as mild and 23, as moderate [69].

Kamgno et al. evaluated and compared side effects of a standard dose and a high dose of ivermectin using a 3-year double-blind RCT. They reported that the common manifestations after ivermectin treatment were itching, edematous swellings, rash, and fever. Also, swellings seemed to be more associated with high-dose treatment as compared to standard dose treatment. In addition to these symptoms which have been reported classically in many literatures, the results showed that treatment with ivermectin was also related with a risk of ocular problems (blurred vision, change in color vision, etc.,) [70]. The pharmacokinetic properties and safety of ivermectin were studied in a multiple-dose clinical trial with healthy volunteers. Sixty-eight volunteers were assigned to four groups: 30 mg (three times a week) followed by a single dose after a 1-week washout, 60 mg (three times a week), 90 mg (single dose), and 120 mg (single dose). Safety assessments addressed both known ivermectin central nervous system (CNS) effects and general toxicity. The primary endpoint of safety was mydriasis, which was precisely quantified by pupillometry. Ivermectin was generally well tolerated, with no associated CNS toxicity at doses up to 10 times the FDA-approved maximum dose of 200 µg/kg. All doses had a mydriatic effect like a placebo. The adverse experiences between ivermectin and placebo were similar and did not increase with the ivermectin dose [32]. A recent meta-analysis showed that adverse events following a single dose treatment of up to 800 µg/kg of ivermectin do not occur with significant differences in frequency or intensity compared to currently approved doses [33]. Some studies reported an increased risk of deaths in elderly patients who were treated with an oral dose of ivermectin (150–200 µg/kg) for scabies. These findings were not replicated in other studies. Besides, long-term studies of ivermectin use in elderly populations found no excess deaths and no serious side effects [71].

The safety of higher doses of ivermectin was investigated in a phase III study that examined 200–400 μg/kg doses in patients with dengue fever and demonstrated that a daily dose of ivermectin treatment over three days is safe [72]. In another study by Guzzo et al., even higher doses (up to 10 times higher than approved doses) were investigated [32]. This study showed that ivermectin was well tolerated during fasting after a one-time dose of 120 mg (10 times higher than the approved dose) and 60 mg three times weekly (every 72 h). In their study, typical side effects of ivermectin treatment include nausea, dizziness, headaches, and rash. The frequency and nature of adverse events were relatively similar between ivermectin (24%) and placebo (35%) and did not increase with increase in the ivermectin dose. All dosages had mydriatic effects (the primary endpoint of safety from toxicology study results) similar to placebos [32]. Similarly, ivermectin was well tolerated at a single dose of 800 µg/kg, at 1600 µg/kg for 12 weeks, and at 1600 µg/kg for 13 days [73]. Also, an oral dose of ivermectin of up to 1400 µg/kg over a month is recommended by the US CDC as a treatment option for scabies [74]. Ocular side effects (temporary blurred vision, itching, pain in the eye, and dyschromatopsia) are important in onchocerciasis patients and require caution and further investigations when ivermectin is used in high doses for this indication. The adverse events reported in the reviewed studies were mostly mild or moderate, suggesting ivermectin’s safety [33].

### Severe side effects of ivermectin

**Fig. 2:**
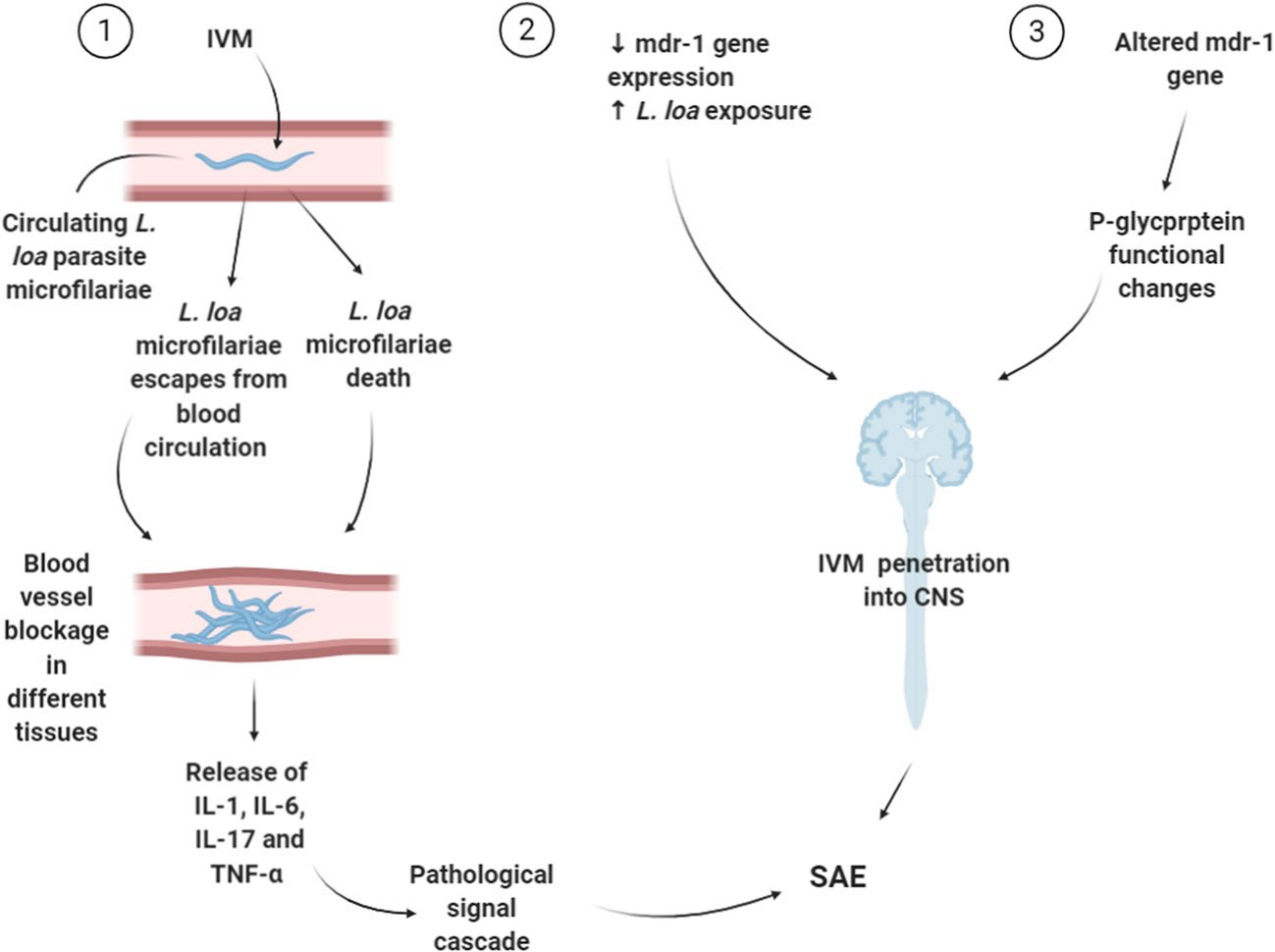
Mechanism of adverse reactions caused by ivermectin in the presence of *L. loa*

In terms of central nervous system-related events, ivermectin is considered safe theoretically, as its distribution to the brain is blocked by the blood–brain barrier (BBB). In particular, this is due to the size of the ivermectin molecule and the presence of efflux pumps. The primary efflux pump that transports ivermectin is the P-glycoprotein pump (P-gp) (ivermectin is both a substrate and a potent inducer of the P-gp). P-gp is involved in transporting ivermectin to the intestinal tract and preventing it from crossing the BBB. Therefore, ivermectin has generally been considered safe and free of potential neurologic adverse drug reactions, except in case of an overdose [75]. However, some neuralgic adverse effects have been reported. In 2018, Chandler et al. reported on the adverse effects from ivermectin and its safety. In their study, the common side effects of the drug were pruritus (25.3%), headache (13.9%), and dizziness (7.5%). They also reported some serious neurological side effects such as encephalopathy and coma [75]. These neurologic side effects were suspected to be due to a CNS infection with a high number of *L. loa* microfilariae and the p-glycoprotein drug pump’s failure as a result of lower expression of the mdr-1 gene [76]. Ivermectin causes the circulatory *L. loa* microfilariae death and their escape into other body fluids. These events result in a blood vessel blockage, which is followed by cytokine and tumor necrosis factor alpha release. The pathological signal cascade is associated with CNS adverse events, as also blocked blood vessels in various tissues cause tissue anoxia and death (Fig. 2). In a study, Twum-Danso et al. assessed all serious adverse events (SAE) that occurred in Africa from the introduction of mass treatment programs with ivermectin for onchocerciasis in 1989 until the end of the calendar year 2001. They reported 207 SAE cases in about 165 million reported treatments performed during the reporting period, resulting in a cumulative incidence of 1 reported SAE per 800,000 reported treatments. 97 cases were encephalopathic, two-thirds of these cases were ‘probable’ or ‘possible’ cases of *Loa loa* encephalopathy, temporally related to ivermectin treatment [77]. In Australia, between January, 1971 and December, 2013, 17 adverse events, including three reports of fatal adverse events from ivermectin treatment were reported to the Therapeutic Goods Administration (TGA), but no causal association with ivermectin could be detected [78]. Table 1 shows the descriptive analysis of adverse events of ivermectin in clinical trials.

**Table 1:** Descriptive analysis of adverse events of ivermectin in clinical trials comparing standard (up to 400 µg/kg) vs. high dose (> 400 µg/kg) of ivermectin

	Condition under study	Ivermectin dosage (µg/kg)	Adverse effects rate (%)	Odds ratio or risk difference (95% CI)
Kamgno et al. [70]	Onchocerciasis			0.96 (0.64–1.44)
	High dose	800	14.8	
	Standard dose	150	15	
Munoz et al. [33]	Healthy volunteers			0.907 (0.369–2.228)
	High dose	401–700	16	
	Standard dose	200–400	17	
Smit et al. [95]	Malaria			6·9% (− 1·9 to 15·7)
	High dose	600	11	
	Standard dose	300	4	
Wimmersberger et al. [33]	Trichuriasis			1.346 (0.532–3.405)
	High dose	600	27	
	Standard dose	100–400	22	

Chandler et al. also reported two deaths attributed to ivermectin [75]. The first was the death of an 81-year-old woman who had received 12 mg of ivermectin on days 0 and 7 for the treatment of acarodermatitis. She died 5 days after the last dose from the events of a depressed level of consciousness and asphyxia. Also, she was receiving other medications like digoxin, rebamipide, crotamiton, and magnesium oxide. The second case was that of a 64-year-old man with a history of giant cellular arteritis treated with prednisone and who had developed sepsis after an aortic valve replacement surgery complicated by multi-system failure. Sputum culture revealed *S. stercoralis* presence. *Strongyloides* *stercoralis* and hyper-infection syndrome was diagnosed, and ivermectin 12 mg was administered every 48 h. He was given three oral doses of the drug, followed by two subcutaneous ones. Despite clinical and microbiological improvements, the patient remained in a vegetative state and died on day 25. The autopsy revealed elevated levels of ivermectin in the brain tissue 14 days after the last dose. Both cases did not meet the criteria of causality of death by ivermectin, but documented the presence of ivermectin in the brain tissue [79]. Sparsa et al. also reported severe side effects of ivermectin at a dose of 200 µg/kg. Most of the side effects observed with this medication were observed in the treatment of two elderly patients with scabies. The first was a 72-year-old man referred for scabies who was treated with benzyl benzoate (Ascabiol) and ivermectin (200 µg/kg) in a single dose. Two days later, the patient had abdominal pain and nausea, and elevated liver function parameters. Laboratory tests did not show cytolysis as a cause of hepatitis. Ivermectin-induced hepatitis was assumed and liver function returned to normal within 2 weeks. The second case was an 86-year-old woman hospitalized for scabies and treated with benzyl benzoate and a single ivermectin dose (200 µg/kg). Three days later, she developed sinus tachycardia and asthma. Ivermectin-induced cardiac toxicity was suspected [80]. Compared to the extensive post-marketing experience with ivermectin, SAEs are rare and have never met the causative criteria. However, clarifying individual risk factors such as advanced age, co-infection, and potential drug polymorphism with mdr-1 gene should be considered [75].

## Discussion

In the light of the pandemic of SARS-COV-2 and the need for new or repurposed medication for the treatment of COVID-19, attention has been paid to three potential anti-viral chemicals. These include anti-malarial (hydroxychloroquine/chloroquine), anti-metabolites (colchicine), [81] and anti-parasitic (ivermectin) [82]. Although initially hydroxychloroquine/chloroquine were thought to be effective agents against SARS-COV-2, subsequent clinical trials have found these agents to be toxic and more harmful than ivermectin. Among these three proposed drugs, ivermectin shows the best safety profile, tolerated at high doses, and the least toxicity profile [33, 46]. Additionally, ivermectin's concurrent use with steroids, the proven effective drugs in the treatment of COVID-19 [83], might be beneficial beyond its potential anti-viral effect in its suppression of parasitic hyper-infection during anti-COVID therapy. Ivermectin targets glutamate-containing chloride channels in invertebrates. However, these agents show selectivity to parasites and do not enter the mammalian central nervous system to cause toxicity [84, 85].

In humans, healthy blood–brain barrier (BBB) and p-glycoproteins typically prevent ivermectin’s entry to the central nervous system and limit its toxicity. However, in patients with hyper-inflammatory diseases, drug–drug interactions can lead to an increase in the concentration of ivermectin, or in case of dysfunctions of the p-glycoprotein pump, ivermectin may penetrate the CNS, and increase the risk of toxicity [86]. Several serious neurological effects of ivermectin are attributed to drug interactions [75], including several cases of the simultaneous use of medicines such as statins, HIV protease inhibitors, calcium channel blockers, and benzodiazepines [75]. Ivermectin is considered to trigger several cytochrome P450 isoenzymes, including CYP1A, 2B, and 3A subfamilies [87], which can disrupt a large percentage of therapeutic agents. Drugs that are substrates of CYP3A4 enzymes are often also substrates of the P-glycoprotein transporter. Therefore, there is a risk of increased absorption beyond the blood–brain barrier when those drug are administered simultaneously with ivermectin [88]. Also, enhanced anti-virals, like lupinavir ritonavir and darunavir are potent cytochrome P450 3A4 inhibitors widely used against SARS-CoV-2. Concomitant use of these drugs with ivermectin may lead to increased systemic exposure to ivermectin and CNS toxicity. Additionally, ritonavir and its enhancers like cobicistat (used to increase the amount of atazanavir) can efficiently inhibit p-glycoprotein, one of the significant outlet pumps in the BBB, thereby causing more neurotoxicity [75]. A recent publication documents evidence of an in vitro interaction between ivermectin and a range of anti-retroviral agents [89]. Current labeling of ivermectin does not warn against its co-administration with CYP3A4 substrates [75]. Available evidence suggests that ivermectin levels with significant activity against SARS-CoV-2 may only be achieved with a considerable increase in its dose which could have toxic effects [84]. Although pharmacokinetic studies on healthy subjects have shown that single doses of up to 120 mg of ivermectin are safe and well-tolerated, the concentration achieved may be of an order of magnitude lower than the Cmax necessary for anti-SARS-COV-2 activity seen in vitro [32]. Some evidence from animal models shows ivermectin levels that are three times more abundant in lung tissue than in plasma, one week after the oral dose [90]. However, even with the high lung:plasma concentrations ratio, ivermectin is unlikely to reach the half-maximal inhibitory concentration (IC50) in the lungs after a single dose of 10 times higher than the approved US Food and Drug Administration (FDA) dose when administered orally [91, 92].

## Conclusion

Based on the current data and the recommended dose of 150–200 µg/kg for COVID-19 treatment, ivermectin is probably safe; however, there is some serious doubt about its efficacy in treating COVID-19. Ivermectin has a better safety profile than other purposed and repurposed drugs such as hydroxychloroquine and colchicine [93] that lack efficacy and, in the case of hydroxychloroquine, has been shown to be harmful [33, 46, 82, 94]. Before initiating a patient on ivermectin therapy, clinicians need to be aware that ivermectin doses necessary to block SARS-COV-2, patients’ inflammatory status, other concurrent medications, and patients' potential genetic polymorphism for the p-glycoprotein mdr-1 gene may enhance ivermectin’s toxicity and serious side effects in humans.

## Data Availability

Not applicable.

## Abbreviations

COVID-19: Coronavirus disease 2019
SARS-CoV-2: Severe acute respiratory syndrome Coronavirus 2
FDA: Food and Drug Administration
GABA: γ-Aminobutyric acid
IFN-I: Type I interferon
JAK: Janus kinase
STAT1: Signal transducer and activator of transcription 1
SAE: Severe adverse events
IMPα/β1: Importin α/β1
NPC: Nuclear pore complex
NF-κB: Nuclear factor-κB
RdRp: RNA-dependent RNA polymerase
HCQ: Hydroxychloroquine
AZT: Azithromycin
BBB: Blood–brain barrier
TGA: Therapeutic Goods Administration
IC50: Inhibitory concentration 50
